# Interspecific formation of the antimicrobial volatile schleiferon

**DOI:** 10.1038/s41598-018-35341-3

**Published:** 2018-11-15

**Authors:** Marco Kai, Uta Effmert, Marie Chantal Lemfack, Birgit Piechulla

**Affiliations:** Institute of Biological Science, University of Rostock, Albert-Einstein-Straße 3, 18059 Rostock, Germany

## Abstract

Microorganisms release a plethora of volatile secondary metabolites. Up to now, it has been widely accepted that these volatile organic compounds are produced and emitted as a final product by a single organism e.g. a bacterial cell. We questioned this commonly assumed perspective and hypothesized that in diversely colonized microbial communities, bacterial cells can passively interact by emitting precursors which non-enzymatically react to form the active final compound. This hypothesis was inspired by the discovery of the bacterial metabolite schleiferon A. This bactericidal volatile compound is formed by a non-enzymatic reaction between acetoin and 2-phenylethylamine. Both precursors are released by *Staphylococcus schleiferi* cells. In order to provide evidence for our hypothesis that these precursors could also be released by bacterial cells of different species, we simultaneously but separately cultivated *Serratia plymuthica* 4Rx13 and *Staphylococcus delphini* 20771 which held responsible for only one precursor necessary for schleiferon A formation, respectively. By mixing their headspace, we demonstrated that these two species were able to deliver the active principle schleiferon A. Such a joint formation of a volatile secondary metabolite by different bacterial species has not been described yet. This highlights a new aspect of interpreting multispecies interactions in microbial communities as not only direct interactions between species might determine and influence the dynamics of the community. Events outside the cell could lead to the appearance of new compounds which could possess new community shaping properties.

## Introduction

Bacterial communities represent very diverse and dynamic systems. This applies especially to densely populated soil, marine and plant- or human-associated habitats where bacterial cells experience a highly competitive environment regarding living space, water and nutrients^1–4^. Survival, growth and flourishing depend on their abilities for quick acclimations to changing living conditions and their strategies to outcompete co-habitants. Consequently, bacteria developed a tight network of interactive patterns consisting of cooperative traits, but also competitive and antagonistic action modes^5^. Antagonism is often realized by the release of secondary metabolites^6^. Research of recent years has shown that many of these secondary metabolites are volatile compounds^7–9^. Because of their properties (small molecules with a high vapor pressure), volatiles do not only easily diffuse into areas close to the emitting organism, but also travel over longer distances. Therefore, they represent antagonistic and/or signalling compounds with a potential to manipulate physiological processes in other bacteria, as well as in fungi and plants^10–14^. The recognition of volatiles enables bacteria to adjust to developmental processes that take place in microbial communities^15^, thereby influencing the motility, biofilm formation and sporulation^16–19^. Furthermore, antibiotic resistances and other stress responses can be enhanced due to contact with bacterial volatiles^18–21^. On the other side, volatile metabolites can exhibit direct bacteriostatic and bactericidal activities^22,23^.

Volatile compounds with such a potential are schleiferons (3-(phenylamino)butan-2-one and 3-(phenylimino)butan-2-one, schleiferon A and B, respectively). They are produced by *Staphylococcus schleiferi* isolates^24^. If present in the environment, schleiferons dramatically decrease the growth of Gram-positive bacteria and affect quorum-sensing controlled phenotypes of Gram-negative bacteria^24^. Schleiferon A is formed by a spontaneous reaction between acetoin and 2-phenylethylamine (Schulz *et al*. unpublished), which are both synthesized by the bacterium *S. schleiferi*. Schleiferon B represents the oxidation product of A (Schulz *et al*. unpublished).

Both acetoin and 2-phenylethylamine are volatile compounds themselves. They are produced by a broad range of bacterial species^25,26^. A bacterial community in general can consist of several different bacterial species. These species *a priori* have the potential to synthesize precursors of final volatile products. This fueled the hypothesis that precursors like acetoin or 2-phenylethylamine released by different species into the headspace of a microbial community will spontaneously react to non-enzymatically form an active principle like schleiferon A (and B). The goal of our experiments was to introduce a mechanism of how new compounds could arise within a habitat colonized by microbes. This mechanism could contribute besides direct microbial interactions to a variable composition of the headspace and adds a new aspect to the in generally known dynamics of microbial communities.

## Results

### The *in vitro* headspace reaction

A preliminary investigation was necessary in order to evaluate whether acetoin and 2-phenylethylamine react in an aerial environment. We separately dropped both compounds in two Petri dishes. These Petri dishes were simultaneously incubated in an analysis chamber for 24 hours. Subsequently, we funneled an air stream through the chamber onto an adsorbent and analyzed the eluted compounds by GC/MS (Fig. S1a). As shown in Fig. S1b, schleiferon A was also formed from acetoin and 2-phenylethylamine when incubated under aerial conditions. Since it lingered in the headspace of the Petri dish, most of it was oxidized to schleiferon B. Consequently, we could search for an acetoin producing bacterial strain that would not be able to emit 2-phenylethylamine and *vice versa* a bacterial isolate that emits solely 2-phenylethylamine.

### Qualified bacterial aspirants

The genus *Serratia* is known to produce acetoin during fermentation of sugars^27^. We analyzed the volatile compounds released by *S. plymuthica* 4Rx13 growing in complex liquid medium supplemented with glucose in a closed dynamic airflow system^24,28^. Already after 24 hours of growth, acetoin (#1) was detected (Fig. S2a). The emission quickly increased during the exponential growth phase and peaked after 48 h of incubation, which corresponded with the early stationary phase of bacterial growth (Fig. S2c). Subsequently, the acetoin level declined and reached the lowest value after 120 hours of growth. Most importantly, 2-phenylethylamine (#2) was not detected at all during these sampling periods.

While screening for a 2-phenylethylamine producer, *S. delphini* 20771 captured our attention. This strain emitted only 2-phenylethylamine during cultivation in tryptic soy broth (TSB) (#2, Fig. S2b). The emission started after 96 h and even increased after 120 hours of growth (Fig S2d). *S. delphini* 20771 did not release acetoin (#1) during these sampling intervals.

### Interspecific bacterial formation of schleiferons

**Figure 1:**
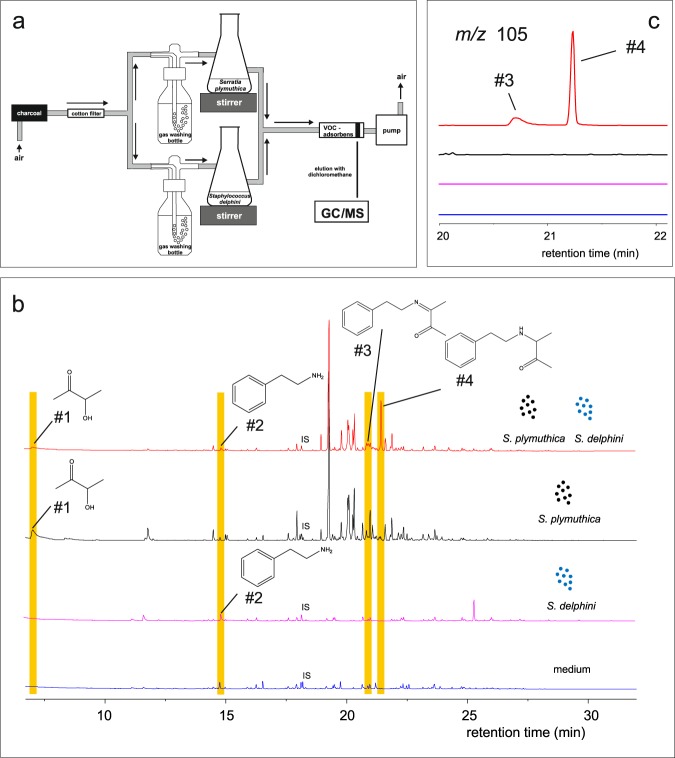
Schleiferon formation in concurrent cultures of physically separated *Serratia plymuthica* and *Staphylococcus delphini*. (**a**) VOC-collection system: Charcoal purified and sterilized air was split into two air streams of which each entered one culture flask containing either *S. plymuthica* 4Rx13 or *S. delphini* 20771. After VOC-enrichment, the two air streams were reunited and funneled over a VOC adsorbent (Porapak) (**b)** TIC-GC/MS chromatograms of media (blue line), *S. delphini* mono-culture (pink line), *S. plymuthica* mono-culture (black line) and *S. plymuthica*/*S. delphini* concurrent cultured for 24 hours (red line) (**c**) EIC-GC/MS chromatograms of *m/z* 105 representing the base peak of schleiferon A and B (color code for experimental set up see (**b**). #1: acetoin, #2: 2-phenylethylamine, #3: schleiferon B, #4: schleiferon A; IS = internal standard (N-nonyl acetate, 5 ng); n = 3.

Based on the results of the mono-cultivation described above, we developed a VOC collection system where both bacterial isolates were concurrently cultivated in separated vessels. A split air stream entered simultaneously one culture flask inoculated with a 24 h old culture of *S. plymuthica* 4Rx13 (flask 1) and one flask with a 120 h old culture of *S. delphini* 20771 (flask 2). The two VOC-enriched air streams were reunited to allow the chemical reaction of acetoin produced by *S. plymuthica* 4Rx13 and 2-phenylethylamine produced by *S. delphini* 20771 (Fig. 1a). We used three controls. In control 1, both flasks were filled with NBII liquid medium supplemented with glucose and TSB, respectively. Sets with one culture flask inoculated with *S. plymuthica* 4Rx13 while the other flask contained TSB and one flask inoculated with *S. delphini* 20771 while the other flask contained NBII plus glucose served as control 2 and 3, respectively. The experiments were performed in triplicate. As expected, the precursor molecule acetoin (#1) was emitted by *S. plymuthica* 4Rx13 (control 2). The precursor 2-phenylethylamine (#2) was present in the VOC profile of *S. delphini* 20771 (control 3). Both controls did not contain schleiferons. Control 1 did not contain precursors nor schleiferons. Small amounts of the precursors were still present in the concurrent culture of both bacterial isolates, however, most interesting was the detection of schleiferon A (#4) and B (#3) in the reunified bacterial headspace of this *S. plymuthica*/*S. delphini* concurrent culture after 24 h and 48 h of cultivation (Fig. 1b). Since *S. plymuthica* 4Rx13 released other compounds co-eluting with schleiferon B (#3), we verified the TIC result by an EIC *m/z* 105 representing the base peak of schleiferon A (#4) and B (#3) (Fig. 1c). The mass feature of *m/z* 105 at the retention time of schleiferon B was unique for the *S. plymuthica*/*S. delphini* concurrent culture.

## Discussion

Our experiments strongly indicate that in a microbial community volatile products can arise from microbial precursor molecules which originate from cells of different species. We used the bacterial isolates *S. plymuthica* 4Rx13 and *S. delphini* DSMZ 20771 as model organisms in order to illustrate the principle. Although these particular isolates did not originate from the same habitat, it can be assumed that acetoin producing bacterial species and 2-phenylethylamine producers can inhabit the same ecological niches. The 2-phenylethylamine producer *Enterococcus faecalis*^29^ and the acetoin producer *Serratia* sp.^30^ both found to be ubiquitous constituents of the gastrointestinal microbiome, represent only one example^31,32^. It should be noted that our *in vitro* experiment required certain nutrient conditions for each strain, in order to provoke the synthesis of the precursor molecules. This situation might ostensibly not be expected when bacteria share the same ecological niche in a natural environment. However, particularly in densely populated microhabitats like soil pores of the rhizosphere or the zone along root hairs, nutrient conditions can be different. Plants secrete metabolites depending on species, age, developmental stage and root zone^33^. Adjacent living cells of different bacterial species might therefore experience different nutrient conditions and accordingly produce a certain spectrum of volatile metabolites, which could undergo a chemical reaction as we could show for acetoin and 2-phenylethylamine. Furthermore, in a populated hotspot where cells of different species mingle even under comparable nutrient supplies, cells of certain species could cause a modification of the ambient milieu in terms of secreting metabolites that serve as nutrient for other species or change conditions (i.e. pH-value) in the microhabitat^34–36^. Those changing conditions can also provoke a changing spectrum of volatile metabolites of certain species. New volatile metabolites emitted into the headspace again could have the potential to initialize chemical reactions. Similar scenarios can be assumed for intestines of animals. A changing pH-value in gut systems and a different status of nutrient digestion might challenge symbiotic gut bacteria in a similar way^37,38^. As those specific alterations in conditions in the microbial habitat might facilitate beneficial chemical reactions, natural environmental conditions, such as water and oxygen saturation, temperature, salinity, chemical composition of the habitat and the pH-value could in general affect headspace reactions. This influence should be questioned in further investigations.

**Figure 2:**
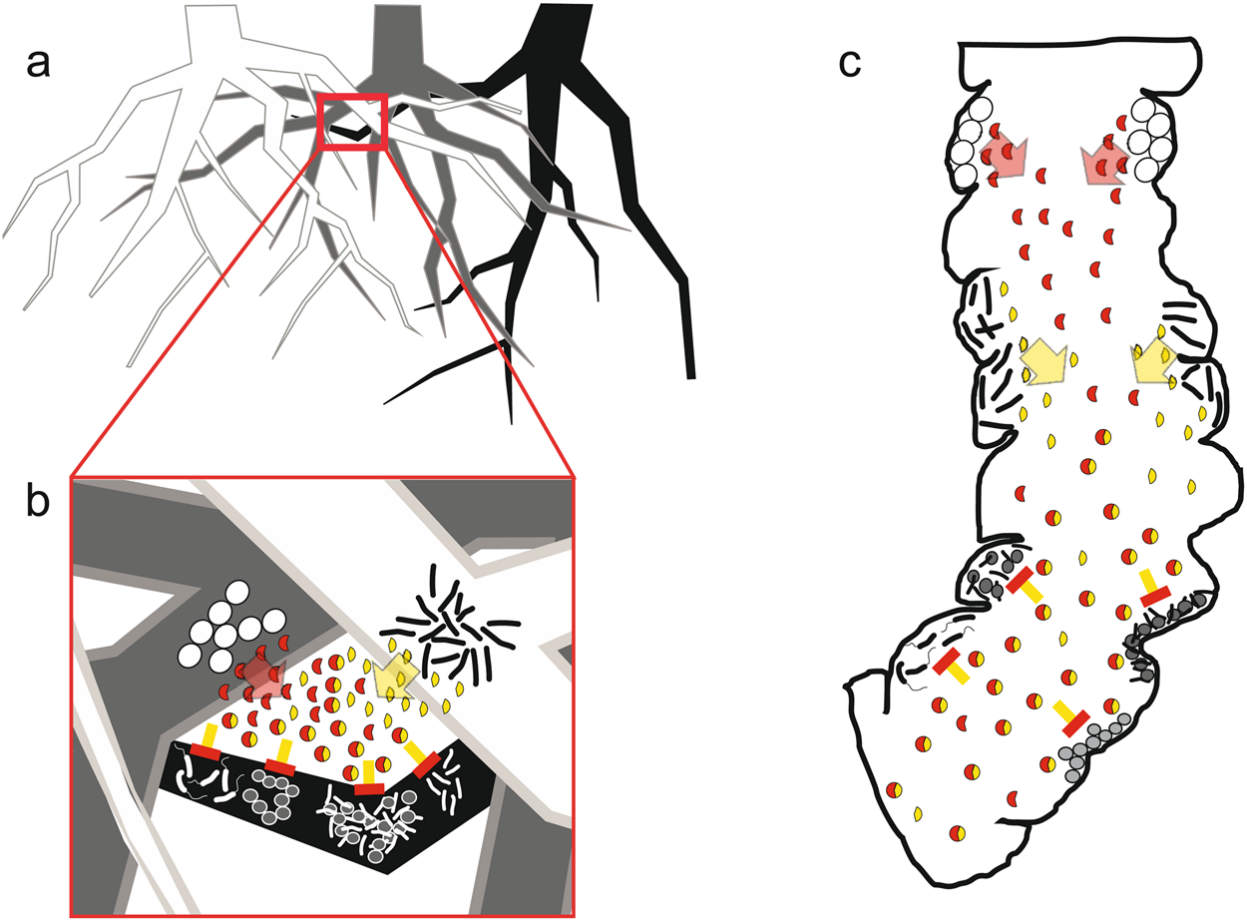
Scenarios of tritrophic interactions between bacterial species mediated by volatile compounds formed by an interspecific reaction. Physically separated bacterial species A and B produce the volatile precursors 1 () and 2 (), respectively. Released into the environment both volatiles react in the airspace of respective habitats as soil pores (**b**) of the rhizosphere (**a**) or the intestine (**c**) forming the active principle (). This compound may impair further microbial organisms e.g. by growth inhibition or manipulation of the quorum sensing-system of bacteria (). Varying nutrient supply due to different root exudates of certain plant species (a and b; roots in white, gray, black) and changing growth conditions as pH-value changes and varying microbial colonization in intestines ensure that a variety of precursors meet and match and furthermore travel to reach a third party which might be harmed. (root drawing: idea from Vecteezy.com^47^).

In conclusion, our results show a new aspect of volatile production in microbial communities. We suggest that not only a single organism i.e. a bacterial cell of a certain species, participates in the synthesis of a final volatile metabolite which is released into the environment. Volatile metabolites could also be formed in the headspace of a microbial community by a non-enzymatic reaction employing precursors that could originate from cells of different species. Consequently, the microbial community itself has to be considered for VOC production. The synthesis of volatile precursors and perhaps even the non-enzymatic reaction might be a result of interaction between species. It is already known that microbial interactions can strongly influence the spectrum of volatiles metabolites emitted by a microbial community^39–43^. However, even if the reaction would be a random result of emitted precursors of accidentally coexisting organisms it could be of the benefit of one or even both producer species. In addition, interspecifically formed volatile secondary metabolites could impair third party organisms i.e. cells of other bacterial or fungal species. Therefore, they could contribute to survival, growth and flourishing of precursor producers in a community (Fig. 2). Producers of matching precursors might seek neighbourly contact for the benefit of both. Since fungi can also form 2-phenylethylamine^44^, inter-kingdom communicative traits can be expected. Hence, the headspace of habitats should not be considered as a static environment but rather as a dynamic system. Although, these conclusions have to be confirmed, they represent an important and non-negligible aspect to describe, evaluate and analyze interactions in a multispecies community and there is plenty of room for further research.

## Methods

### Bacterial strains and maintenance

The rhizobacterium *Serratia plymuthica* 4Rx13 was isolated from *Brassica napus*^45^. *Staphylococcus delphini* DSMZ 20771 originated from purulent material from a dolphin^46^. Short-term maintenance of *S. plymuthica* 4Rx13 was performed on nutrient agar plates supplemented with glucose (NAIIG; peptone from casein 3.5 g l^−1^, peptone from meat 2.5 g l^−1^, peptone from gelatine 2.5 g l^−1^, yeast extract 1.5 g l^−1^, NaCl 5 g l^−1^, glucose 100 mM, agar-agar 15 g l^−1^). *S. delphini* was cultivated on tryptic soy agar plates (TSA; tryptone 17 g l^−1^, soy meal 3 g l^−1^, K_2_HPO_4_ 2.5 g l^−1^, glucose 2.5 g l^−1^, NaCl 5 g l^−1^, agar-agar 15 g l^−1^). Cultures were incubated for 12–24 hours at 30 °C under dim light (1.5 µEm^−2^s^−1^) and finally stored at 4 °C. For conservation, overnight liquid cultures were supplemented with glycerol (25%) and subsequently stored at − 70 °C.

### Reaction of acetoin and 2-phenylethylamine in an aerial environment

Acetoin (200 µg dissolved in 1 ml dichloromethane) and 2-phenylethylamine (400 µg dissolved in 1 ml dichloromethane) were separately dropped in a glass Petri dish (Ø30 × 10 mm). These Petri dishes were placed without a lid in another glass Petri dish (Ø130 × 30 mm), which had an in- and outlet (analysis chamber). After 24 h of incubation (30 °C and 1.5 µEm^−2^s^−1^), charcoal-purified, sterile air was sucked through the inlet into the analysis chamber with a constant flow of 0.6 l min^−1^ provided by a membrane pump (1410 Büh 12 VDC “D”, Gardner Denver Thomas GmbH, Memmingen, Germany). The volatile-enriched air was directed to a trap containing 30 mg of an adsorbent matrix (Porapak; Sigma–Aldrich, Munich, Germany). After 24 h of incubation, the volatiles were consecutively eluted with 200 and 100 µl dichloromethane. N-nonyl acetate solution was added as an internal standard (final concentration of 5 ng µl^−1^).

### VOC collection of mono-cultivated bacterial strains

A single bacterial colony of *S. plymuthica* 4Rx13 and S. *delphini* DSMZ 20771 was inoculated into 6 ml nutrient broth II supplemented with glucose (NBIIG) and tryptic soy broth (TSB), respectively. Cultures were incubated overnight under agitation (160 rpm; Bühler, Tübingen, Germany) at 30 °C until reaching an OD_600_ of 1–2. They were diluted into 100 ml medium (NBIIG or TSB) to obtain an initial OD_600_ of 0.005 and further incubated for 120 h using specific Erlenmeyer flasks supplied with an in- and outlet nozzle (30 °C, 1.5 µEm^−2^s^−1^, agitation using a magnetic stirrer). Pure medium served as control. VOC collection was performed as described in Kai *et al*. 2010 and Lemfack *et al*. 2016^24,28^. Charcoal-purified, sterile and humidified air was sucked through the inlet into the flask with a constant flow of 0.6 l min^−1^ provided by a membrane pump (1410 Büh 12 VDC “D”, Gardner Denver Thomas GmbH, Memmingen, Germany). The volatile-enriched air was directed into a trap containing 30 mg of an adsorbent matrix (Porapak; Sigma–Aldrich, Munich, Germany). After defined incubation intervals (24, 48, 72, 96 and 120 h), the volatiles were consecutively eluted with 200 and 100 µl dichloromethane. N-nonyl acetate was added as an internal standard (final concentration of 5 ng µl^−1^). Simultaneously, the living cell number was determined at every 24 h by serial dilution in NaCl-solution (0.8%). The dilutions were plated and CFUs were counted. The experiments were conducted in triplicate.

### VOC collection of physically separated concurrently cultivated bacterial strains

The pre-cultivation of the bacterial strains and inoculation into the Erlenmeyer flasks was performed as described above. The VOC-collection system was modified as followed. Charcoal-purified and sterile air was sucked with a constant flow of 0.6 l min^−1^ (1410 Büh 12 VDC “D”, Gardner Denver Thomas GmbH, Memmingen, Germany). After passing the cotton filter, the air stream was split, humidified and directed through two Erlenmeyer flasks (Fig. 1a). While one Erlenmeyer flask was filled with a liquid culture of *S. plymuthica* 4Rx13 (24 h post inoculation), the second flask contained a *S. delphini* 20771 culture (120 h post inoculation). Three additional setups served as control. Flask 1 and 2 contained NBII + glucose and TSB, respectively (control 1). Furthermore, a mono-culture of *S. plymuthica* 4Rx13 in flask 1 was combined with TSB in flask 2 (control 2) and the mono-culture of *S. delphini* 20771 in flask 2 was combined with NBII + glucose in flask 1 (control 3). Cultures were magnetically stirred. Volatile-enriched air streams were reunited and sucked into a trap containing 30 mg of an adsorbent matrix (Porapak; Sigma–Aldrich, Munich, Germany). VOCs were consecutively eluted after 24 h and 48 h of cultivation with 200 and 100 µl dichloromethane. N-nonyl acetate was added as an internal standard (final concentration of 5 ng µl^−1^). The experiments were conducted in triplicate.

### Gas-chromatographic separation and mass spectrometric detection of acetoin, 2-phenylethylamine and schleiferons

Samples were analyzed using a Shimadzu GC/MS-QP5000 system (Kyoto, Japan). Separation was performed on a DB5-MS column (60 m × 0.25 mm × 0.25 μm; J&W Scientific, Folsom, California, USA) connected to a CTC autosampler (CTC Analytics, Zwingen, Switzerland). The sample (1 µl) was splitless injected at 200 °C with a solvent cut of 2 minutes. The initial temperature of the GC column was set at 35 °C. Compounds were separated using a continuous ramp of 10 °C min^−1^ up to 280 °C with a final hold of 5 minutes. Helium was used as carrier gas (flow rate of 1.1 ml min^−1^, linear velocity of 28 cm/s).

Electron ionization mass spectra (EIMS) were recorded at an ionization energy of 70 eV with a mass range of m/z 40–280. Schleiferon A and B were identified by comparison of mass spectra and retention times with those of synthesized standards.

## Electronic supplementary material

SUPPLEMENTARY INFORMATION
