# Publisher Correction: Interspecific formation of the antimicrobial volatile schleiferon

**DOI:** 10.1038/s41598-018-37081-w

**Published:** 2019-02-26

**Authors:** Marco Kai, Uta Effmert, Marie Chantal Lemfack, Birgit Piechulla

**Affiliations:** Institute of Biological Science, University of Rostock, Albert-Einstein-Straße 3, 18059 Rostock, Germany

Correction to: *Scientific Reports* 10.1038/s41598-018-35341-3, published online 15 November 2018

The original PDF version of this Article contained additional symbols superimposed above Fig. 2a which were erroneously introduced during the Production process. This has now been corrected in the PDF; the HTML version of the paper was correct from the time of publication.

