# Supplementary material for: Interspecific formation of the antimicrobial volatile schleiferon
Source: Sci Rep. 2018 Nov 15;8:16852. doi: 10.1038/s41598-018-35341-3 (PMC6237861; doi:10.1038/s41598-018-35341-3)
Supplement: Supplementary file 1 — SUPPLEMENTARY INFORMATION [file 41598_2018_35341_MOESM1_ESM.docx]

**Interspecific formation of the antimicrobial volatile schleiferon**

Marco Kai*, Uta Effmert, Marie Chantal Lemfack, Birgit Piechulla

Institute of Biological Science, University of Rostock, Albert-Einstein-Straße 3, 18059 Rostock, Germany

**Figure S1**: Schleiferon formation in an aerial environment a) Reaction system – acetoin and 2-phenylethylamine were simultaneously incubated in an analysis chamber for 24 hours. Subsequently, an air stream was directed through the chamber onto an adsorbent and eluted compounds were analyzed by GC/MS b) TIC-GC/MS chromatogram – formation of #3: schleiferon B, #4: schleiferon A. Small amount of: 2-phenylethylamine (#2) is still present, while acetoin was not detected, IS: internal standard (N-nonyl acetate, 5 ng µl^−1^)

**Figure S2**: VOC-analysis of mono-cultivated *Serratia plymuthica* 4Rx13 and *Staphylococcus delphini* 20771. a) TIC-GC/MS chromatogram of headspace volatiles of *S. plymuthica* 4Rx13. Accumulation between 0 and 24 h. b) TIC-GC/MS chromatogram of headspace volatiles of *S. delphini* 20771. Accumulation between 96 and 120 h. #1: acetoin, #2: 2-phenylethylamine, IS: internal standard (N-nonyl acetate, 5 ng µl^−1^) c) relative emission of #1 acetoin (grey columns) and growth of *S. plymuthica* 4Rx13 during sampling intervals (dashed line). d: relative emission of #2 2-phenylethylamine (grey columns) and growth of *S. delphini* 20771 during sampling intervals (dashed line).
